# Fluid sparing effects of chemokine (C-C motif) receptor 1 and 2 antagonists during resuscitation from hemorrhagic shock in rat models

**DOI:** 10.1371/journal.pone.0351212

**Published:** 2026-06-11

**Authors:** Elizabeth A. Cook, Ololade Ogunsina, Xianlong Gao, Matthias Majetschak

**Affiliations:** 1 Department of Surgery, Morsani College of Medicine, University of South Florida, Tampa, Florida, United States of America; 2 Department of Molecular Pharmacology and Physiology, Morsani College of Medicine, University of South Florida, Tampa, Florida, United States of America; Scuola Superiore Sant'Anna, ITALY

## Abstract

We described previously that the chemokine (C-C motif) receptor 1 (CCR1) and CCR2 antagonists BX471 and INCB3284 reduce fluid requirements during resuscitation after hemorrhage. Their effects, however, have not been directly compared with each other and consequences of simultaneous blockade of CCR1/2 are unknown. Here we utilized rat (Sprague-Dawley) models of hemorrhagic shock to compare fluid sparing properties when administered individually or in combination and to assess effects on shock tolerance. Series 1: rats were hemorrhaged to a mean arterial blood pressure (MAP) of 30 mmHg for 30 min, followed by blood pressure-directed fluid resuscitation for 6h. At t = 30 min, vehicle (n = 12), BX471 (0.5 µmol/kg, n = 9), INCB3284 (5 µmol/kg, n = 7), or BX471 (0.5 µmol/kg) plus INCB3284 (5 µmol/kg, n = 6) were injected. Series 2: rats were hemorrhaged to a MAP of 30 mmHg for 45 min, injected with vehicle (n = 8), BX471 (0.5 µmol/kg, n = 7) or INCB3284 (5 µmol/kg, n = 5), and observed until t = 225 min. Series 1: cumulative fluid requirements sharply increased between t = 220–300 min and averaged 108±20 mL/kg at t = 390 min with vehicle-treatment. After treatment with BX471, INCB3284 or both, fluid requirements remained constant and averaged 31±8 mL/kg, 51±12 mL/kg and 36.5±8 mL/kg, respectively, at t = 390 min (p < 0.05 vs. vehicle). Mortality was 75% with vehicle treatment and 33%, 57% and 66% with BX471, INCB3284 and BX471 plus INCB3284 treatment, respectively (p > 0.05 vs. vehicle). Measurements of a panel of systemic inflammation markers suggested that BX471 and INCB3284 attenuate release of TNFα and IL6, and enhance release of CCL5 during resuscitation. Series 2: BX471 and INCB3284 treatment did not affect survival times. Our findings confirm fluid sparing effects of BX471 and INCB3284 over 6h of resuscitation, suggest that both drugs exert comparable efficacy to reduce fluid requirements and modulate the systemic inflammatory response to hemorrhage and fluid resuscitation.

## Introduction

In the United States trauma is the leading cause of death up to the age of 50 years and hemorrhagic shock remains a major cause of preventable death after injuries [1,2]. While pre-hospital fluid resuscitation is essential for the treatment of hemorrhagic shock when blood components are not available, the risks of under- and over-resuscitation are well recognized. The ideal fluid resuscitation strategy and the choice of resuscitation fluid, however, are still a matter of debate [3]. Moreover, drugs that could be used to improve fluid resuscitation after hemorrhagic shock are not available. Such drugs would provide the potential to significantly reduce morbidity and mortality after traumatic-hemorrhagic shock.

Chemokines and their receptors are known to play a critical role in inflammation, regulating functions including cell migration, cell proliferation, tissue repair, and angiogenesis [4]. With numerous shared ligands between chemokine receptors creating redundancy in the system, the efficacy and translation to clinical practice of monotherapies targeting specific chemokines and/or their receptors have been challenging [5].

Chemokine (C-C motif) receptor 1 (CCR1) and CCR2 are evolutionary closely related receptors and share multiple chemokine ligands, many of which are known to contribute to the regulation of the inflammatory response after trauma and hemorrhage [6–10]. CCR1 antagonism has been studied and found to be beneficial in preclinical models of inflammatory processes such as sepsis, acute pancreatitis, and transplant organ rejection [11–13]. The CCL2/CCR2 axis has similarly been identified as a potential therapeutic target in both acute and chronic processes such as ischemia-reperfusion injury, cardiovascular disease, and cancer associated inflammation [4,5,14].

Recently, we identified CCR1 and CCR2 as drug targets to improve fluid resuscitation after hemorrhagic shock [9,10,15]. We showed that the selective CCR1 antagonist BX471 as well as the selective CCR2 antagonist INCB3284 significantly reduce fluid requirements to maintain hemodynamics in rat models of hemorrhagic shock [9,10,15]. The effects of BX471 and INCB3284 after hemorrhagic shock, however, have not been directly compared with each other, and the consequences of simultaneous blockade of CCR1 and CCR2 during fluid resuscitation after hemorrhage are unknown. In addition, potential effects of BX471 on shock tolerance have not been evaluated. Thus, in the present study, we utilized rat models to compare fluid sparing effects of BX471 and INCB3284 during resuscitation after hemorrhagic shock, to evaluate the effects of co-administration of BX471 and INCB3284, and to test whether each chemokine receptor antagonist modulates shock tolerance after hemorrhage in the absence of fluid resuscitation.

## Materials and Methods

### Drugs

INCB3284 and BX471 were purchased from Tocris, Bio-Techne Corporation (Minneapolis, MN, USA).

### Hemorrhagic Shock Models

All procedures were performed in accordance with the National Institutes of Health Guidelines for use of Laboratory Animals and were approved by the Institutional Animal Care and Use Committee (IACUC) of the University of South Florida under protocol number IS00012148. The IACUC specifically reviewed and approved the anticipated mortality in the study design. Male Sprague-Dawley rats weighing between 330 and 400g were obtained from Envigo (Indianapolis, IN, USA). General anesthesia was induced utilizing an isoflurane-soaked gauze and a bell jar. The animals were subsequently transferred to the procedural area where sedation was maintained using nosecone inhalation at a level of 2.6% isoflurane delivered by the SomnoSuite small animal anesthesia system (Kent Scientific Corporation, Torrington, CT, USA). Throughout the procedure, sedation was maintained at a level such that the animal was not responsive to noxious stimuli while still maintaining spontaneous respirations. The left femoral artery and right femoral vein were isolated utilizing a direct cutdown method and cannulated with a 24-gauge and 26-gauge catheter, respectively. The arterial line was used for continuous hemodynamic monitoring and as an access point for controlled hemorrhage. The venous line was used for drug and intravenous fluid administration. Following cannulation, isoflurane was reduced to 1.7% and the animal monitored for a period of 10 minutes to ensure stability before proceeding. Hemodynamics were monitored continuously throughout the experiment using the Surgivet invasive blood pressure monitor (Med-Electronics, Beltsville, MD, USA) and recorded at two-minute intervals during hemorrhage and five-minute intervals during the resuscitation or observation periods. In all experiments animals were continuously monitored and remained under general anesthesia until euthanasia or death, as defined by cessation of spontaneous respirations and MAP ≤ 15 mmHg in all experiments.

### Resuscitation series

Rats underwent controlled hemorrhage via the arterial line to a mean arterial blood pressure (MAP) of 30 mmHg for 30 min. At the end of the hemorrhage period (t = 30 min), control animals (n = 12) received lactated ringer’s solution (=vehicle) delivered in 1 mL boluses via the femoral venous line until either systolic blood pressure (SBP) recovered to 90 mmHg or MAP recovered to 60 mmHg. Additional 0.5–1 mL boluses were administered as needed to maintain this blood pressure goal for the duration of the experiment. Experimental animals were hemorrhaged in an identical fashion and received either a 1 mL intravenous injection of 0.5 µmol/kg of BX471 (n = 9), a 5 µmol/kg dose of INCB3284 (n = 7), or a combination dose of 0.5 µmol/kg of BX471 plus 5 µmol/kg of INCB3284 (n = 6) followed by additional LR boluses as needed to maintain hemodynamic goals. INCB3284 was dissolved in normal saline to a stock solution of 10 mM and further diluted in phosphate buffered saline to the required doses. BX471 was dissolved in phosphate buffered saline to a stock solution of 10 mM and further diluted in phosphate buffered saline to the required doses. The doses of BX471 and INCB3284 were selected based on our previous studies [9,10]. Animal experiments were performed in an alternating order, starting with vehicle, BX471 and INCB3284 treatment groups, and adding the BX471 plus INCB3284-treatment group alternating with vehicle-treated animals later. For all groups, the experiment was concluded at the end of a 360-minute resuscitation period or at the time point the animal expired. Death was defined as cessation of spontaneous respirations and MAP ≤ 15 mmHg. Blood samples were collected for blood gas and laboratory analysis at the beginning of hemorrhage (t = 0 min), at the end of hemorrhage (t = 30 min), midway through resuscitation (t = 210 min), and at resuscitation end (t = 390 min). Serum samples were collected at t = 0 min and at the end of the experiment in animals who survived at least until t = 330 min. Serum samples were stored at −80°C for subsequent analysis. At the end of the experiment (t = 390 min) or when death criteria had been met, animals were euthanized (5% isoflurane, bilateral pneumothorax, ventriculotomy).

### Terminal hemorrhage series

Rats were hemorrhaged to a MAP of 30 mmHg for 45 min. At the end of this period, control animals (n = 8) received a single 1 mL bolus of vehicle while experimental animals received either 0.5 µmol/kg BX471 (n = 7) or 5 µmol/kg INCB3284 (n = 5) delivered in a 1 mL bolus of vehicle. No additional fluid was administered in any group. Hemodynamics were observed continuously and recorded for a period of 180 minutes or until the animal expired, as defined above.

### Arterial Blood Gases and Laboratory Parameters

Arterial blood gases, lactate, hemoglobin and hematocrit were measured using the Element point of care veterinary blood gas, electrolyte, and critical care analyzer (Cuattro Veterinary USA, Loveland, CO, USA).

### Enzyme-linked immunosorbent assay (ELISA)

Multiplex ELISA was performed measuring relative concentrations of tumor necrosis factor α (TNFα), interleukin 6 (IL6), interferon γ (IFNγ), IL1α, IL-1β, chemokine (C-C motif) ligand 2 (CCL2, also known as monocyte chemoattractant protein 1, MCP-1), CCL3 (also known as macrophage inflammatory protein 1α, MIP-1α) and CCL5 (also known as RANTES) in serum samples following manufacturer instructions using the commercially available Rat Inflammation ELISA Colorimetric Strip (Signosis, Santa Clara, CA, USA). Relative concentrations of the analytes were expressed as optical density (OD) measured at λ 450nm/mg of protein.

### Protein Measurements

Total protein concentrations in serum samples were measured with the Thermo Scientific NanoDrop One Spectrophotometer (Waltham, MA, USA) according to the manufacturer’s instructions.

### Data analyses and statistics

The GraphPad Prism program version 10.3.1 (GraphPad Software, San Diego, CA, USA) was utilized for all data analyses. Data are presented as mean ± SE and were analyzed with 2-way analysis of variance (ANOVA) with Holm-Šídák’s multiple comparisons test. The ROUT method with a maximum false discovery rate of 1% was applied to physiologic outcomes to detect outliers. Survival data were plotted as Kaplan-Meier curves and analyzed using the log-rank test. A two-tailed p < 0.05 was considered significant.

## Results

**Fig 1:**
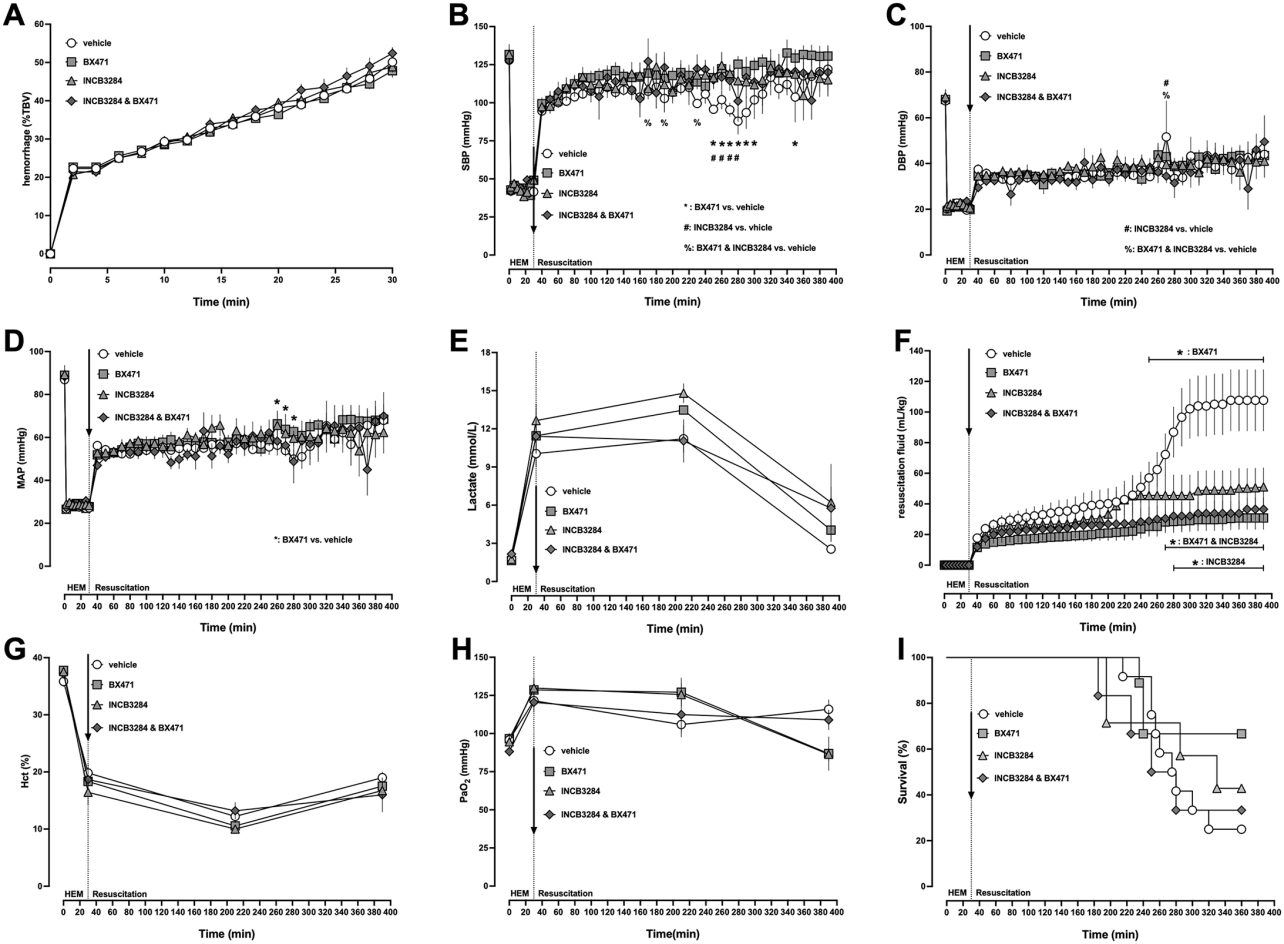
Effects of BX471 and INCB3284 during resuscitation after hemorrhage. Animals were hemorrhaged to a MAP of 30 mmHg for 30 min followed by 360 min of fluid resuscitation. Animal received either vehicle (open circles, n = 12), 0.5 µmol/kg of BX471 (grey squares, n = 9), 5 µmol/kg dose of INCB3284 (grey triangles, n = 7), or a combination dose of 0.5 µmol/kg of BX471 plus 5 µmol/kg of INCB3284 (grey diamonds, n = 6). Arrow indicates vehicle/drug administration and start of the resuscitation period. HEM: hemorrhage period. Data are mean ± SE (Fig 1A-H) or survival percentages (Fig 1I). *, % and # denote p < 0.05 vs. vehicle treated animals, as indicated in the graphs. **A**. Blood volume hemorrhaged as percentage of total blood volume (%TBV). **B.** Systolic blood pressure (SBP, mmHg). **C**. Diastolic blood pressure (DBP, mmHg). **D.** Mean arterial blood pressure (MAP, mmHg). **E**. Lactate concentrations (mmol/L). **F**. Cumulative resuscitation fluid requirements (mL/kg). **G**. Hematocrit (%). **H**. PaO_2_, partial pressure of oxygen in arterial blood (mmHg). **I.** Survival (%).

To evaluate and compare effects of BX471 and INCB3284 after hemorrhagic shock, animals were treated with a single intravenous injection of 1 mL of vehicle, 0.5 µmol/kg of BX471, 5 µmol/kg of INCB3284, or with a combination of 0.5 µmol/kg of BX471 plus 5 µmol/kg of INCB3284, at the beginning of fluid resuscitation. There were no differences in any of the physiological parameters among the groups at the beginning of the experiments. The hemorrhage volumes to maintain a MAP of 30 mmHg during the shock period were comparable among all groups (Fig 1A). Fig 1B-D show the average SBP, diastolic blood pressure (DBP) and MAP, respectively, during the entire observation period. As compared with vehicle treated animals, systemic blood pressures of the animals in the treatment groups showed only short lasting and transient elevations throughout the resuscitation period. In all animals, blood lactate concentrations increased from baseline to 10–12.6 mmol/L at the end of the shock period and declined in surviving animals during subsequent fluid resuscitation (Fig 1E). Blood lactate concentrations were indistinguishable among the groups (p > 0.05 vs. vehicle for all treatment groups).

The cumulative resuscitation fluid requirements to maintain hemodynamics are shown in Fig 1F. In vehicle-treated animals, cumulative fluid requirements to maintain hemodynamics sharply increased between t = 220 min to t = 300 min and averaged 108 ± 20 mL/kg at the end of the observation period. Cumulative fluid requirements at the end of the observation period averaged 31 ± 8 mL/kg in animals treated with BX471, and 51 ± 12 mL/kg and 36.5 ± 8 mL/kg in animals treated with INCB3284 and in animals treated with BX471 plus INCB3284, respectively (p < 0.05 vs. vehicle treated animals for all treatment groups). The resuscitation fluid volume (mL) to hemorrhage volume (in mL of blood) ratios (R/H-ratios) averaged 3.5 ± 0.6 in vehicle-treated animals, and 1.0 ± 0.2, 1.6 ± 0.4 and 1.1 ± 0.2 after BX471, INCB3284 and BX471 plus INCB3284 treatment, respectively (p < 0.05 vs. vehicle for all). There were no statistically significant differences in cumulative fluid requirements or R/H-ratios among the treatment groups. Hematocrit values (Fig 1G) and PaO_2_ (Fig 1 H) were comparable among all experimental groups. Mortality was 75% with vehicle treatment and 33%, 57% and 66% with BX471, INCB3284 and BX471 plus INCB3284 treatment, respectively (Fig 1I, p > 0.05 vs. vehicle treatment for all). The median survival time was 277.5 min with vehicle treatment, undefined with BX471 treatment, and 330 min and 265 min with INCB3284 and BX471 plus INCB3284 treatment, respectively (p > 0.05 among all groups).

**Fig 2:**
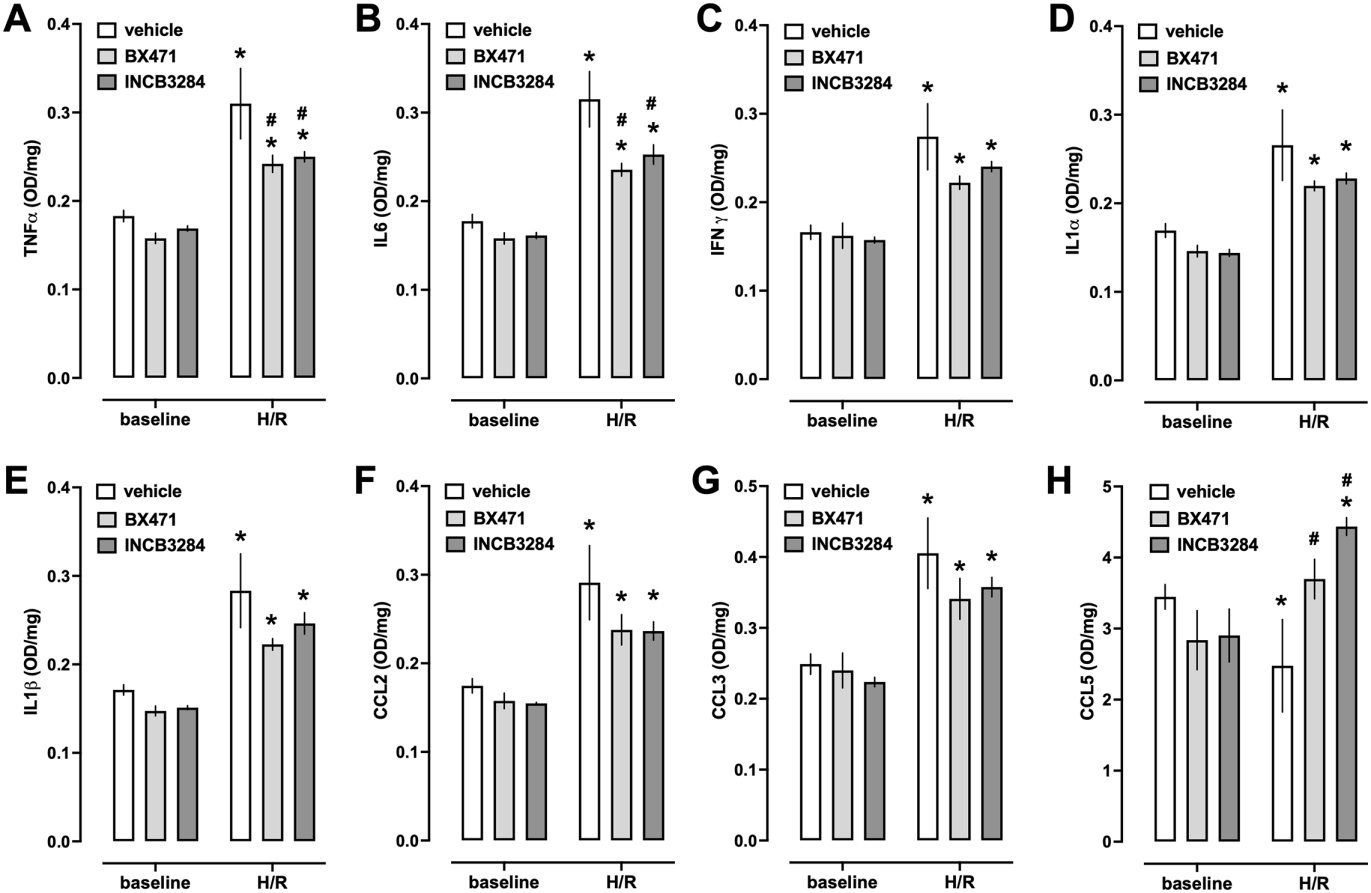
Effects of BX471 and INCB3284 on serum markers of inflammation. Serum markers of inflammation were measured at t = 0 min (baseline) and following hemorrhage and resuscitation (H/R) in animals surviving greater than 5h. Open bars: vehicle treated animals, n = 5. H/R samples were obtained at t = 390 min (n = 3), t = 350 min (n = 1) and t = 330 min (n = 1). Light grey bars: BX471 treated animals, n = 5. All H/R samples were obtained at t = 390 min. Dark grey bars: INCB3284 treated animals, n = 4. H/R samples were obtained at t = 390 min (n = 3) and t = 360 min (n = 1). Inflammation marker concentrations are expressed as OD per mg of total protein (OD/mg). Data are mean ± SE. *: p < 0.05 for baseline vs. H/R. #: p < 0.05 for H/R vehicle vs. H/R BX471 or H/R INCB3284. **A**. TNFα. **B**. IL6. **C**. IFNγ. **D**. IL1α. **E**. IL1β. **F**. CCL2. **G**. CCL3. **H**. CCL5.

To assess whether BX471 or INCB3284 modulate the systemic inflammatory response to hemorrhage and fluid resuscitation, we measured the concentrations of a panel of serum inflammation markers at baseline and at the end of the experiments in animals that survived at least until t = 330 min (Fig 2). Serum from animals after treatment with BX471 plus INCB3284 was not analyzed because only two animals survived longer than t = 330 min. Baseline concentrations per mg of total protein of the inflammation markers were comparable in vehicle-, BX471- and INCB3284-treated animals (p > 0.05 vs vehicle treatment for all). As compared with baseline concentrations, serum levels of TNFα, IL6, IFNγ, IL1α, IL1β, CCL2 and CCL3 significantly increased during fluid resuscitation after hemorrhage in all animals (Fig 2A-G). When compared with vehicle-treated animals, serum concentrations of TNFα and IL6 during resuscitation were significantly lower in BX471 and INCB3284 treated animals (Fig 2A,B). While serum concentrations of IFNγ, IL1α, IL1β, CCL2 and CCL3 during resuscitation were also lower in BX471 and INCB3284 treated animals, as compared with vehicle-treated animals, these differences did not reach statistical significance. In contrast, serum concentrations of CCL5 decreased during resuscitation with vehicle treatment but increased with BX471 and INCB3284 treatment (Fig 2H).

**Fig 3:**
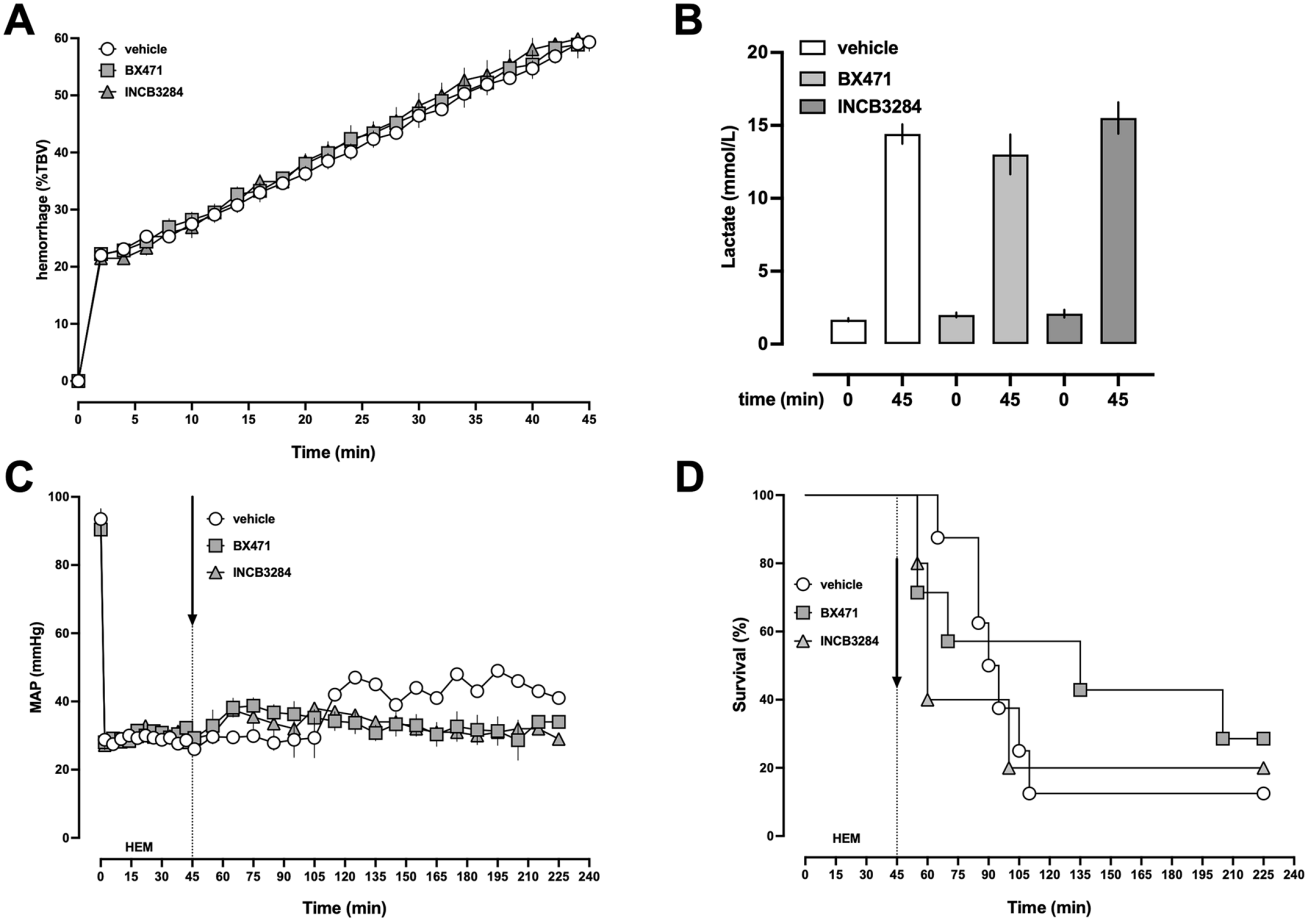
Effects of BX471 and INCB3284 on shock tolerance. Animals were hemorrhaged to MAP of 30 mmHg for 45 min followed by an observation period of 180 min. At t = 45 min, animals received either vehicle (n = 8, open circles/bar), 0.5 µmol/kg BX471 (n = 7, light grey squares/bar) or 5 µmol/kg INCB3284 (n = 5, dark grey triangles/bar) without further intervention. Arrows indicate time of vehicle/drug administration. HEM: hemorrhage period. Data are mean ± SE (A-C) or percentage survival **(D)**. **A**. Blood volume hemorrhaged as percentage of total blood volume (%TBV). **B**. Lactate concentrations at t = 0 and t = 45 min. **C**. Mean arterial blood pressure (mmHg). **D**. Survival (%).

To evaluate and compare effects of BX471 and INCB3284 on shock tolerance in the absence of fluid resuscitation, animals were hemorrhaged to a MAP of 30 mmHg for 45 min, injected with 1 mL of vehicle, 0.5 µmol/kg of BX471 or 5 µmol/kg of INCB3284, and then observed until t = 225 min without further intervention (Fig 3). Two animals died during the hemorrhage period before vehicle or drug injection. The hemorrhage volumes to maintain MAP at 30 mmHg for 45 min (Fig 3A) and increases in blood lactate concentrations during the hemorrhage period (Fig 3B) were comparable among all groups. As shown in Fig 3C, MAP did not recover spontaneously during the observation period. With vehicle treatment, 1 out of 8 animals survived the observation period and the median survival time was 92.5 min (Fig 3D). Two out of 7 animals treated with BX471 and 1 out of 5 animals treated with INCB3284 survived the observation period (Fig 3D). The median survival times were 135 min with BX471 treatment and 60 min with INCB3284 treatment. Survival proportions and median survival times were not significantly different between the groups.

## Discussion

In the present study, we continued to characterize and compare the effects of BX471 and INCB3284 in rat models of hemorrhagic shock. As documented by increases in lactate concentrations to greater than 10 mmol/L at the end of the hemorrhage periods, the development of pronounced fluid requirements during resuscitation and significant mortality during the observation periods, the rat hemorrhage models in the present study reflect severe hemorrhagic shock. We previously reported that administration of 0.5 µmol/kg BX471 and of 5 µmol/kg INCB3284 provide fluid sparing effects during resuscitation after 30 min of hemorrhage to a MAP of 30 mmHg when animals were resuscitated for up to 3 hours and 4.5 hours, respectively [9,10]. The findings of the present study confirm our previous observations and suggest that such fluid sparing effects of the same doses of BX471 and INCB3284 are detectable during resuscitation periods of up to 6 hours. Although cumulative fluid requirements were lowest in animals treated with BX471, either when administered alone or in combination with INCB3284, the slightly higher cumulative fluid requirements with INCB3284 treatment were not significantly different. Thus, our findings suggest that pharmacological blockade of CCR1 and of CCR2 during resuscitation after hemorrhage exert fluid sparing effects of similar magnitude. In addition, our findings do not support the assumption that blockade of both chemokine receptors provides additional benefits in our experimental model. However, administration of BX471 reduced fluid requirements by approximately 70%, as compared with vehicle treated animals. Because the volume of resuscitation fluid was equal to the hemorrhage volume in BX471-treated animals, any further reduction of fluid requirements may not be possible, which may have masked potential synergistic or additive effects of co-administration of BX471 and INCB3284. Thus, experimental models in which the effects of the chemokine receptor antagonists are less pronounced may be required to further evaluate whether dual targeting of CCR1 and CCR2 could improve therapeutic efficacy.

Despite the profound reduction in fluid requirements by BX471 and INCB3284, there were no significant differences in survival times and survival proportions between the groups. Given the observed mortality proportions of 75% with vehicle treatment, 33% with BX471 treatment and 57% with INCB8234 treatment, we calculate that 21 and 108 animals in each group, respectively, would be required to detect a significant survival benefit with BX471 or INCB3284 treatment compared to vehicle treated animals with alpha = 0.05 and a power of 80%. Thus, the present resuscitation study, which was designed to evaluate and compare fluid sparing effects of the chemokine receptor antagonists, was underpowered to detect differences in mortality.

The observations that hematocrit values, lactate concentrations and PaO_2_ were indistinguishable among the groups suggest that right heart failure due to acute fluid overload or hypoxemia are unlike to account for mortality, which point toward loss of vascular tone with subsequent circulatory failure as a reason for mortality, a common event during resuscitation from hemorrhagic shock [16].

Furthermore, our findings suggest that blockade of CCR1 and of CCR2 modulate the magnitude of the release of several inflammatory molecules which contribute to the complex inflammation cascades that regulate organ function during shock phases. These single-time point observations, however, are limited in scope and unable to provide a detailed assessment of the release kinetics of the measured inflammation markers. For example, we reported previously that systemic CCL5 concentrations were significantly elevated in vehicle-treated animals in the same experimental model after 30−60 min of resuscitation [10]. Thus, the finding that CCL5 concentrations were decreased in vehicle-treated animals after 6h of resuscitation in the present study could be explained by transient increases in CCL5 levels during early resuscitation periods with subsequent decreases of CCL5 levels after longer time periods. Nevertheless, our findings that BX471 and INCB3284 reduced hemorrhage and resuscitation-associated increases in TNFα and IL-6 concentrations and enhanced CCL5 concentrations support the concept that these drugs modulate the systemic inflammatory response during resuscitation after hemorrhage. Furthermore, due to the small number of surviving animals that were treated with both chemokine receptor antagonists, we cannot comment on inflammation marker concentrations after dual receptor blockade. Because the main outcome parameter in the present study was reduction of fluid requirements, which achieved statistical significance at the current sample size, we abstained from increasing the number of animals in the co-treatment group to adhere to the 3R guidelines of animal experimentation [17].

In contrast to the fluid sparing effects of BX471 and INCB3287 in the resuscitation model, both chemokine receptor antagonists did not affect survival times after hemorrhage in the absence of fluid resuscitation. These observations are consistent with our previous findings on the effects of INCB3284 in a model that mimicked continuous hemorrhage without fluid resuscitation and indicate that both drugs do not influence shock tolerance [15].

While our study was designed to compare fluid sparing effects of BX471 and INCB3287 during resuscitation after hemorrhage and to assess their effects on shock tolerance, the present study is unable to address the potential mechanisms underlying their fluid sparing properties. Nevertheless, the finding that both drugs reduced fluid requirements while hematocrit values were indistinguishable from vehicle-treated animals suggests reduction of third-spacing of fluids and points toward modulation of endothelial function as one potential mechanism. Such a mechanism would be consistent with previous findings suggesting that CCR1 and CCR2 contribute to the development of vascular leakiness and edema formation in disease processes [18–20]. Alternatively, both chemokine receptors have been described to modulate vascular smooth muscle responsiveness to stress hormones, which may also have contributed to the observed effect [9,21,22]. Because CCR1 and CCR2 are closely related chemokine receptors that share multiple endogenous ligands, similar mechanisms underlying their fluid sparing effects appear likely.

## Conclusion

The present study confirms fluid sparing effects of BX471 and INCB3284 in rat models of hemorrhagic shock and fluid resuscitation. Our findings demonstrate that such effects are detectable at least over 6 hours of resuscitation, indicate that both drugs exert fluid sparing effects of similar magnitude, and suggest that blockade of CCR1 and CCR2 modulate the systemic inflammatory response during resuscitation after hemorrhage. We believe that our observations justify further evaluation of the therapeutic potential of CCR1 and CCR2 as drug targets to improve fluid resuscitation in conditions associated with hemorrhagic shock.
